# Evaluating the Prevalence and Predictors of Moderate to Severe Depression in Fort McMurray, Canada during the COVID-19 Pandemic

**DOI:** 10.3390/ijerph19127090

**Published:** 2022-06-09

**Authors:** Gloria Obuobi-Donkor, Ejemai Eboreime, Reham Shalaby, Belinda Agyapong, Folajinmi Oluwasina, Medard Adu, Ernest Owusu, Wanying Mao, Vincent I. O. Agyapong

**Affiliations:** 1 Department of Psychiatry, University of Alberta, Edmonton, AB T6G 2B7, Canada; obuobido@ualberta.ca (G.O.-D.); eboreime@ualberta.ca (E.E.); rshalaby@ualberta.ca (R.S.); bagyapon@ualberta.ca (B.A.); folajinm@ualberta.ca (F.O.); medard@ualberta.ca (M.A.); eowusu2@ualberta.ca (E.O.); wmao2@ualberta.ca (W.M.); 2 Global Psychological E-Health Foundation, Edmonton, AB T6G 2B7, Canada; 3 QEII Health Sciences Centre, Department of Psychiatry, Faculty of Medicine, Dalhousie University, Halifax, NS B3H 2E2, Canada

**Keywords:** Major Depressive Disorder, COVID-19, mental health, employer support, Fort Mcmurray

## Abstract

Background: The Coronavirus disease (COVID-19) pandemic has produced adverse health consequences, including mental health consequences. Studies indicate that residents of Fort McMurray, a community which has experienced trauma from flooding and wildfires in the past, may be more vulnerable to the mental health effects of the pandemic. Objective: This study aimed to examine the prevalence and predictors of likely Major Depressive Disorder (MDD) among residents of Fort McMurray during the COVID-19 pandemic. Methods: A cross-sectional approach was adopted utilizing an online survey questionnaire to gather sociodemographic data, COVID-19 related data, and clinical information, including likely MDD using the Patient Health Questionnaire (PHQ-9) scale, from the residents of Fort McMurray between the period of 24 April to 2 June 2021. Results: Overall, 186 individuals completed the survey out of 249 residents who accessed the online survey, yielding a completion rate of 74.7%. The prevalence of likely MDD among respondents was 45%. Respondents willing to receive mental health counselling were five times more likely to experience MDD during the COVID-19 pandemic (OR = 5.48; 95% CI: 1.95–15.40). Respondents with a history of depression were nearly five folds more likely to report MDD during the era of the pandemic than residents without a history of depression (OR = 4.64; 95% CI: 1.49–14.44). Similarly, respondents with a history of taking hypnotics (sleeping tablets) were nearly six-fold more likely to express MDD than respondents with no history of receiving sleeping tablets (OR = 5.72; 95% CI: 1.08–30.30). Finally, respondents who reported receiving only partial support from the employer had three times higher odds of having likely MDD than those who received absolute support from the employer (OR = 3.50; 95% CI: 1.24–9.82). Conclusion: In addition to the effect of the pandemic and other measures taken to curb the psychopathological impact of the pandemic, policymakers need to implement policies to manage individuals with preexisting mental health conditions and provide strong employer support.

## 1. Introduction

Since the World Health Organization (WHO) announced the Coronavirus disease (COVID-19) as a public health emergency of international concern on 30 January 2020, the infection has become one of the significant threats to global public health [1]. COVID-19 has taken millions of lives globally; as of 22 March 2022, 470,839,745 people had been confirmed positive for COVID-19 globally, with an estimated 6,092,933 deaths [2]. In Canada, 3,397,593 people had been confirmed positive for COVID-19 as of the same date, with 37,169 deaths [2].

The emergence of the pandemic has been associated with mental health and socioeconomic consequences and the rate of likely depression symptoms increased among individuals [3]. For example, a study in Italy conducted by Fiorillo et al. revealed that out of the 20,720 participants studied, 2555 reported severely or extremely severe depression symptoms during isolation due to the COVID-19 pandemic [4]. Some depression symptoms include nearly every day experiencing diminished ability to think or concentrate, fatigue, insomnia, or hypersomnia [5]. Individuals affected by the virus experience stigma that adversely affects their psychological wellbeing. The widespread fear caused by the COVID-19 pandemic, and the stringent measures to control it, are potential mental health stressors [6]. Traceable to COVID-19, authorities worldwide implemented policies to curtail the disease. Such implementations include lockdowns, vaccines, quarantine, and isolation measures, which adversely affect the mental health of most individuals [4,7,8]. Researchers have documented the mental health effect on individuals in times of crisis. A cross-sectional study in South Asia recorded a higher prevalence of depression among 82.4% of the respondents examined due to the pandemic [9].

Another cross-sectional study conducted in India during the COVID-19 pandemic revealed a prevalence rate of 25% of moderate to extremely severe depression [10]. Similarly, a study conducted in Canada showed one week prevalence rate for self-reported moderate to high stress, Generalized Anxiety Disorder (GAD), and likely MDD as 84.9%, 46.7%, and 41.4%, respectively [11]. Furthermore, another study conducted in Alberta during the pandemic reported a prevalence of 44.0% of likely MDD among respondents [12].

The increasing infection rate and traumatic death of individuals took a toll on health workers and patients. Healthcare workers are psychologically affected by the pandemic as well. For example, research conducted in China to compare medical workers with the general population during the pandemic revealed that 23.13% of medical workers in China reported depression symptoms since the pandemic [13]. Likewise, a meta-analysis showed a 45% pooled prevalence of depression among COVID-19 patients [14]. Similarly, in Canada, a study to assess the impact of COVID-19 among nurses showed a 5% increase in anxiety and depression from 10% to 15% pre-pandemic and early stages of the pandemic, respectively [15]. These studies demonstrate the pandemic’s psychological impact; and the need to treat any mental health condition that emerges, considering that untreated depression can be complicated with suicidal ideation, social dysfunction, and severe cognitive impairment [16,17].

Previous studies have shown that various factors can contribute to adverse mental health during the pandemic and other traumatic events. These predictors include sociodemographic characteristics, preexisting mental health diagnoses (e.g., depression), and social support, including governmental and family and friends, support received during the pandemic [18,19]. Furthermore, preexisting physical illness [20], living in urban areas [21], individuals with the confirmed or suspected COVID-19 infection together with their family or friends, occupational exposure risks [22] and weak immune system are some determinants of mental health illnesses [3].

Although various studies make contributions during the pandemic, important questions remain regarding the factors influencing moderate to severe depression symptoms amid the pandemic in Fort McMurray. The study aims to report depression symptoms among residents of Fort McMurray and identify risk factors for moderate to severe depression among residents of Fort McMurray during the COVID-19 pandemic. Further, given that the Fort McMurray community has experienced previous natural disasters in the past five years, our study uniquely throws light on how the ongoing pandemic may have affected the mental health of residents of a vulnerable community.

## 2. Methodology

### 2.1. Study Setting

Fort McMurray is in the Northern part of Alberta, Canada. The municipality has a population of 111,687 as of 2018 [23]. Residents of Fort McMurray have experienced a series of traumatic events in recent times, such as the 2016 wildfire that destroyed homes and forced many residents out of their homes [24], and the 2020 flooding [25]. These traumatic events affected the mental health of the residents. A study by Agyapong et al. shows a one-month prevalence rate for likely MDD, GAD and Post Traumatic Stress Disorder (PTSD) as 19.8%, 12.8% and 14.8%, respectively, six months after the Fort McMurray wildfire [24,26,27]. The COVID-19 potentially adds an extra psychological burden on residents of Fort McMurray. Following the pandemic, Albertans (including residents of Fort McMurray) received support from the Government and other entities. Such support includes, but has not been limited to, food, medical supply, and financial support [28].

### 2.2. Study Design

This study was a cross-sectional survey conducted between 24 April and 2 June 2021. Multistage sampling approach was used. The first stage was a convenience sampling of intermediaries through which respondents could most easily be reached at Fort McMurray. These intermediaries include the government, schools, occupational and community platforms. The questionnaires were distributed randomly via email to residents of Fort McMurray via these intermediaries. Data were collected using online questionnaires administered via REDCap [29]. This approach was necessitated by the COVID-19 pandemic restrictions and was approved by the ethics committee.

### 2.3. Outcomes and Measures

The survey measured likely MDD among residents using the Patient Health Questionnaire (PHQ-9), which scores each of the nine DSM-IV criteria as “0” (not at all) to “3” (nearly every day) [30]. The PHQ-9 scale categorizes depression based on scores into none-minimal (0–4 points), mild (5–9 points), moderate (10–14 points), moderately severe (15–19 points), and severe (20–27 points). The scores were recategorized into a binary variable: a) none to mild depression and b) moderate to severe depression. The scale used to assess depression symptoms was self-reported, hence, the likely depression symptoms.

The justification for this was based on the recommendations for active management. Typically, some form of active management of depression is recommended for patients scoring above 10 points, whereas no active treatment is recommended for patients with none to mild depression [30]. In addition, the survey contained questions related to respondents’ mental health and medication history, as well as exposure to COVID-19 pandemic news. Data were also collected on the support received from family and friends, employers, the Government of Alberta, and Canada during the pandemic.

### 2.4. Statistical Analysis

Data were analyzed using Statistical Package for Social Sciences (SPSS) version 25 (IBM Corp 2011, Armonk, NY, USA) [31]. Demographic, clinical and other variables were described, and sex analysis was conducted. Cross-tabular analyses using the Chi-square test explored relationships, categorical predictors, and the likelihood that respondents self-reported MDD during the COVID-19 pandemic.

Multivariate binary logistic regression analysis was examined to determine predictor variables for respondents to self-report moderate to severe symptoms of depression during the COVID-19 pandemic, controlling for the other variables. From the bivariate analysis, the variables with a statistically significant or near significant association (*p* ≤ 0.1) with the likelihood of respondents to self-report moderate to severe depression, were included in the regression model. The multivariate logistic regression analysis was run after correlational analysis was performed to exclude any strong intercorrelations (Spearman’s correlation coefficient of 0.7 to 1.0 or −0.7 to −1.0) among predictor variables. Odds ratios (OR) and confidence intervals (C.I) were reported. There was no imputation for missing data, and the data analyzed and reported reflect the number of responses for each question.

### 2.5. Ethical Considerations

Participants were provided with information about the survey, and informed consent was implied by completing the survey questionnaires. The University of Alberta Health Research Ethics Committee approved this study (Pro00066054).

## 3. Results

Two hundred and forty-nine individuals accessed the online survey link, out of which 186 completed the survey, giving a response rate of 74.7%. Descriptive characteristics of the sample of respondents (*n* = 186) were examined against the sex at birth of the participants (Table 1). As shown in Table 1, most respondents 98 (52.7%) were above 40 years of age; 175 (94.1%) were employed; 132 (71.0%) were married or partnered; 58 (31.2%) reported history of depression while 90 (48.4%) reported having no mental health diagnosis; 59 (31.7%) were on antidepressants; 120 (64.5%) reported not receiving psychotropic medications; 72 (38.7%) reported receiving mental health counselling in the past, and 98 (52.7%) said they would like to receive mental health counselling. With reference to COVID-19 related variables, 160 (92%) of respondents reported having been fearful about contracting the virus; 168 (96.6%) were afraid about their close friends or family members contracting the Coronavirus; 124 (72.1%) reported having close friends or family members who have been sick from the coronavirus disease; 104 (60.1%) had to self-isolate or self-quarantine due to COVID-19 symptoms, recent travel, or being in contact with someone who may have COVID-19; 77 (44.3%) watched television images of sick and dead people caused by Coronavirus, on a daily basis; 101 (58%) of respondent daily read newspaper and internet articles related to the pandemic; 153 (87.9%) reported not losing their job due to the COVID-19 pandemic; Concerning social support received since the COVID-19 pandemic declared, respondents who received absolute support from family and friends were 76 (43.9%), those who reported no support from the Government of Canada were 92 (54.4%); while 107 (62.9%) reported no support from the Government of Alberta. Similarly, 78 (45.3%) of respondents reported receiving absolute support from the employer. The prevalence of moderate to severe depression was 45.0% during the COVID-19 pandemic in Fort McMurray.

The bivariable analysis in Table 2 included thirty-two variables: demographic, clinical and COVID-related association with the likelihood of MDD. The chi-squared or Fisher exact test showed a significant (*p* ≤ 0.05) association between the likelihood of MDD and sixteen variables: employment status; history of depression; history of anxiety diagnosis; history of any mental health diagnosis; history of antidepressant medications; history of benzodiazepine medications; history of sleeping tablets, history of receiving any psychotropic medications; receiving mental health counselling; willingness to receiving mental health counselling; lost job due to the COVID-19 pandemic; fearful of close friends or family members contracting COVID-19; receiving support from family or friends; receiving support from the Government of Alberta; receiving support from the employer since the COVID-19 pandemic was declared. Those who received support from the Government of Canada showed a near significant (0.1 < *p* < 0.05) association with likely MDD.

### Multivariate Binary Logistic Regression Results

From Chi square analysis, there were fifteen variables that showed significance and one that neared significance. After running correlation analysis, we had eleven variables eligible for the regression model, after excluding variables that showed a high correlation (collinearity) with other variables and variables that showed low variability.

Table 3 represents the multivariate logistic regression model to ascertain the association between independent (clinical and COVID-19 related) variables and the MDD variable among respondents in Fort McMurray.

The entire model containing all predictors was statistically significant; *Χ^2^* (df = 17; *n* = 164) = 72.26, *p* < 0.001, indicating that the model was able to distinguish between respondents who have moderate to severe depression and those who are at most have a mild depression during the COVID-19 pandemic at Fort McMurray. The model explained the variance between 35.6% (Cox and Snell R^2^) to 47.6% (Nagelkerke R^2^). According to the goodness-of-fit statistic using Hosmer-Lemeshow goodness-of-fit test, the model was adequately fit (Chi^2^ = 7.15; *p* = 0.52). The model correctly classified 76.8% of cases.

As shown in Table 3, only four independent variables made a unique statistically significant contribution to the model (history of depression, history of taking sleeping tablets, willingness to receive mental health counselling and support from the employer).

The strongest association was found among respondents willing to receive mental health counselling. These respondents had five times greater odds of having likely MDD during the pandemic than those who did not require mental health counselling (OR = 5.48; 95% CI: 1.95–15.40). Similarly, respondents who had MDD in the past were nearly five times more likely to have likely MDD (OR = 4.64; 95% CI: 1.49–14.44) than respondents with no history of MDD diagnosed by a health professional. Again, respondent who received partial support from the employer had more than three folds higher odds than those who received absolute support (OR = 3.495; 95% CI 1.244–9.818). Finally, respondents who were currently taking sleeping tablets had nearly sixfold greater odds of having likely MDD during the pandemic when compared to respondents who were not taking sleeping tablets (OR = 5.72; 95% CI: 1.08–30.30).

The logistic regression model results show that job loss, support from family and friends, and support from the government during the pandemic had no statistically significant association with the likelihood for respondents to present with likely MDD.

## 4. Discussion

The prevalence of likely MDD among our respondents was 45%. Our findings agree with other studies showing an increase in depression symptoms following the pandemic. A national survey conducted in Canada to ascertain depression before and during COVID-19 in 2021 showed an increase from 4% to 10% respectively, since the onset of COVID-19 [32]. Prevalence varies in population, sample size, place, time, and even the type of traumatic event [33]. For example, six months after the 2018 wildfire in Fort McMurray, the prevalence of MDD was 19.8% among residents [24] and 24.8% eighteen months after the same traumatic event [34]. These rates are lower than the prevalence of MDD during the pandemic recorded at 45% in this study.

Similarly, various studies have recorded higher rates of MDD during the COVID-19 pandemic, which is consistent with our research. For example, a study in British Columbia, Canada, showed a prevalence of 15% during the pandemic [15].

Our study did not find a significant association between receiving support from family and friends and the presence and severity of depression. This finding is counterintuitive given that empathy and the support of loved ones are expected to allay potential fears and distress in times of crisis, as demonstrated in existing research. For example, a study conducted in China showed that family support is a protective factor for developing mental health conditions and improves psychological health during the COVID-19 pandemic [35]. Similarly, Liu et al. found that family support reduces stress on individuals during the pandemic and increases resilience to health problems such as depression [36]. However, given that over 50% of the respondents in our study have a history of mental health conditions and may be receiving support from family and friends, there is the possibility of a responder bias dissociating the existing support from support received during the pandemic.

Some governmental bodies have supported people since the emergence of the pandemic. The Government of Alberta supported residents with financial assistance and encouraged working remotely to curtail the spread and protect the health of individuals [28]. An evidenced-based study in the United States indicated that Governmental aid mitigates psychological stress and reduces depression [37]. The Government of Canada provided health tips to deal with depression during the pandemic and emergency financial aid, which reduced stress [38]. Government support did not significantly impact respondents’ likelihood of moderate to severe depression. However, individuals may have received benefits directly from their employer or received Governmental support indirectly through the employer; thereby, the individual does not appreciate a direct positive impact of the Governmental support.

Further, losing jobs during the pandemic had no statistically significant association with the presence or severity of depression symptoms. Being employed or obtaining a job provides individuals with confidence, improves self-esteem, and brings control with life and emotions [39]. Notwithstanding, this finding contrasts with previous studies that associate job loss with a high risk of developing poor mental health during disasters [40]. For example, a survey conducted among 723 participants to establish the relationship between job loss and mental health during the pandemic revealed that participants who lost their job had increased incidence and severity of depression symptoms [39]. Given the meagre unemployment rate (6%), the low incidence of job loss during the pandemic (12%), and the associated wide confidence interval, this lack of association should be interpreted with caution. However, studies involving a larger sample size may clarify this finding.

### 4.1. Covariates and Potential Effect Modifiers

Covariates and potential effect modifiers in this study include pre-existing diagnoses of depression, the willingness to receive mental health counselling and a history of taking hypnotics (sleeping tablets).

Our study revealed that respondents with a history of depression were nearly five times more likely to report MDD during the COVID-19 pandemic. Our result is consistent with other studies, which showed that participants who had a pre-existing diagnosis of depression from a health professional were more likely to exhibit depression symptomatology [4,34,41,42]. For example, results from the COMET collaborative network showed that people with pre-existing mental health conditions have a greater risk of experiencing severe depression symptoms (*p* < 0.0001) [4]. This may be explained by people who have a well-established diagnosis of mental health conditions being in a poorer position to deal with the stress during a crisis such as COVID-19 than those without pre-existing mental health problems. A prior diagnosis of depression supports the idea that existing mental health diagnosis exposes one to further mental health trauma and suggests that an individual with a diagnosis of depression may be more prone to developing severe conditions when faced with crises.

Respondents willing to receive mental health counselling were five times more likely to express depression symptoms. This could be explained by individuals with mental health conditions wanting to receive mental health counselling to cope with their conditions and vice versa. This agrees with other literature. Studies examining traumatic events and mental health have shown that respondents willing to receive mental health counselling are at risk of expressing mental health symptoms such as depression [34] while other studies stipulated that counselling after a traumatic experience may not be advantageous to the individual [43], especially when it is a single session of counselling [44]; mental health counselling in the pandemic era was proved to help reduce depression symptoms [45].

Respondents with a history of taking sleeping tablets were almost six times more likely to express depression symptoms. This concerns a study by the Holmquist research group, which shows that most respondents with insomnia and depression symptoms resort to sleeping tablets [46]. A survey by Tsuno and Ritchie to examine the relationship between sleep disturbance and depression revealed that excessive use of sleeping tablets could explain the pathophysiology of depression symptoms [47]. This study is also consistent with our research which suggested that respondents on sleeping tablets are likely to express depression symptoms. Again, individuals with sleep disturbance are more likely to become dependent on hypnotics, which may be associated with depression symptoms. Most studies have established a relationship between sleep deprivation, sleeping tablets (example, triazolam) and experiencing depression symptoms [48,49]. Furthermore, a clinical survey conducted to ascertain sleep disturbance and likely depression found that 25 of the 33-sample population receiving sleeping tablets expressed depression symptomatology [48].

### 4.2. Policy Implications and Future Directions

Psychological first aid can be a choice of intervention during or after traumatic events. This enhances adaptive coping, and reduces acute stress and depression symptoms [50]. Governmental and employer support can improve resilience and further prevent depression symptoms. Hence the Government of Canada and Alberta, together with employers, should provide adequate support to citizens. Furthermore, policies can adopt novel electronic and mobile health options which are economical and effective to minimize mental health illnesses. A study by Agyapong et al. concluded that daily supportive text messaging could reduce psychological distress in the wake of COVID-19 [18,51,52]. The same research team revealed that, after three months of subscribing to the Text4Hope program, subscribers reported a 10.3% reduction in moderate to high depression symptoms compared to baseline data [51]. The study period enabled the researchers to assess the mental health impact of residents of Fort McMurray since the pandemic was at its peak. Treating pre-existing mental health conditions and providing effective counselling can reduce further psychological stress and improve resilience. Further studies are needed to ascertain other variables that may contribute to moderate to severe depression symptoms in the era of COVID-19 and other traumatic events. In-depth research is needed into the association between sleeping tablets and likely depression symptoms during pandemics.

### 4.3. Limitations of This Study

Our study has some limitations which need to be considered when interpreting our findings. First, we relied on online convenience sampling methods since the pandemic did not allow for a more systematic approach to data collection. Thus, our findings may not represent the general population of Fort McMurray. However, our sample may be considered to reflect the residents of Fort McMurray who were accessible during the pandemic through the government and institutional intermediary channels used. Further, the MDD measurement scale (PHQ-9) used in the study was self-reported by residents, thus should not be interpreted as an objective clinical diagnosis of MDD or its severity. The PHQ-9 is however considered an excellent screening tool for depression disorders in primary care [53]. Finally, due to the large number of variables and the lack of correction for multiple testing in our regression model, our study outcome may be prone to type 1 error. On the other hand, routine use of corrections methods such as Bonferroni correction have been criticized as deleterious to sound statistical judgment and reduce the chance of a type I error at the expense of a type II error [54]. Notwithstanding these limitations, the current results add to other COVID-19 studies and the limited literature on predictors and estimates of likely MDD during the pandemic.

## 5. Conclusions

This study establishes the prevalence and associated factors of MDD symptomatology among residents of Fort McMurray during the pandemic. The study results suggest that a history of pre-existing diagnosis of depression, willingness to receive mental health counselling, receiving some support from an employer and taking sleeping tablets contribute to likely depression symptoms. Policymakers need to mitigate the threat of the pandemic and incorporate effective and accessible supportive messaging to prevent and reduce moderate to severe symptoms of depression among Fort McMurray residents and improve quality of life.

## Figures and Tables

**Table 1:** Descriptive Characteristics of the Sample.

Variables	Male *n* (%)	Female *n* (%)	Overall *n* (%)
**Age categories**			
≤25 years	4 (14.8)	9 (5.7)	13 (7.0)
<26–40 years	5 (18.5)	70 (44.0)	75 (40.3)
>40 years	18 (66.7)	80 (50.3)	98 (52.7)
**Employment status**			
Employed	24 (88.9)	151 (95.0)	175 (94.1)
Unemployed	3 (11.1)	8 (5.0)	11 (5.9)
**Marital Status**			
Married/partnered	17 (63.0)	115 (72.3)	132 (71.0)
Divorced/Separated	1 (3.7)	17 (10.7)	18 (9.7)
Single	9 (33.3)	27 (17.0)	36 (19.4)
**mental health diagnosis**			
Depression	6 (22.2)	52 (32.7)	58 (31.2)
Bipolar Disorder	1 (3.7)	5 (3.1)	6 (3.2)
Anxiety	9 (33.3)	69 (43.4)	78 (41.9)
Schizophrenia	0 (0.0)	0 (0.0)	0 (0.0)
Personality Disorder	0 (0.0)	2 (1.3)	2 (1.1)
Other diagnoses	5 (18.5)	12 (7.5)	17 (9.1)
No mental health diagnosis	15 (55.6)	75 (47.2)	90 (48.4)
**History of psychotropic medications**			
Antidepressants	7 (25.8)	52 (32.7)	59 (31.7)
Antipsychotics	3 (11.1)	1 (0.6)	4 (2.2)
Benzodiazepines	0 (0.0)	4(2.5)	4 (2.2)
Mood stabilizers	3 (11.1)	9 (5.7)	12 (6.5)
Sleeping tablets	4 (14.8)	17 (10.7)	21 (11.3)
Other	1 (3.7)	2 (1.3)	3 (1.6)
Not on medication	18 (66.7)	102 (64.2)	120 (64.5)
**Mental Health counselling in the past year**			
No	18 (66.7)	96 (60.4)	114 (61.3)
Yes	9 (33.3)	63 (39.6)	72 (38.7)
**Willingness to receive mental health counselling**			
No	18 (66.7)	70 (44.0)	88 (47.3)
Yes	9 (33.3)	89 (56.0)	98 (52.7)
**Fearful about contracting the coronavirus**			
No	7 (29.2)	7 (4.7)	14 (8.0)
Yes	17 (70.8)	143 (95.3)	160 (92.0)
**Fearful about close friends or family members contracting the coronavirus**			
No	2 (8.3)	4 (2.7)	6 (3.4)
Yes	22 (91.7)	146 (97.3)	168 (96.6)
**Close friends or family members sick from the coronavirus disease**			
No	3 (13.0)	45 (30.2)	48 (27.9)
Yes	20 (87.0)	104 (69.8)	124 (72.1)
**Self-isolate or self-quarantine due to COVID-19 symptoms, recent travel, or in contact with someone who may have COVID-19**			
No	12 (50.0)	57 (38.3)	69 (39.9)
Yes	12 (50.0)	92 (61.7)	104 (60.1)
**Frequency watching television images of sick and dead people caused by a coronavirus**			
Daily	12 (50.0)	65 (43.3)	77 (44.3)
Less than daily	10 (41.7)	64 (42.7)	74 (42.5)
Not at all	2 (8.3)	21 (14.0)	23 (13.2)
**Frequency reading newspaper and internet articles related to the pandemic**			
Daily	14 (58.3)	87 (58.0)	101 (58.0)
Less than daily	9 (37.5)	60 (40.0)	69 (39.7)
Not at all	1 (4.2)	3 (2.0)	4 (2.3)
**Lost your job due to the COVID-19**			
No	22 (91.7)	131 (87.3)	153 (87.9)
Yes	2 (8.3)	19 (12.7)	21 (12.1)
**Received sufficient support from family and friends since the COVID-19**			
Absolute support	10 (41.7)	66 (44.3)	76 (43.9)
Some support	10 (41.7)	45 (30.2)	55 (31.8)
Limited support	1 (4.2)	25 (16.8)	26 (15.0)
No support	3 (12.5)	13 (8.7)	16 (9.2)
**Received sufficient support from**			
**Government of Canada since the COVID-19**			
Absolute support	3 (13.0)	19 (13.0)	22 (13.0)
Some support	3 (13.0)	24 (16.4)	27 (16.0)
Limited support	3 (13.0)	25 (17.1)	28 (16.6)
No support	14 (60.9)	78 (53.4)	92 (54.4)
**Received sufficient support from**			
**Government of Alberta since the COVID-19**			
Absolute support	3 (13.0)	13 (8.8)	16 (9.4)
Some support	3 (13.0)	22 (15.0)	25 (14.7)
Limited support	1 (4.3)	21 (14.3)	22 (12.9)
No support	16 (69.6)	91 (61.9)	107 (62.9)
**Received sufficient support from**			
**Employer since the COVID-19**			
Absolute support	9 (37.5)	69 (46.6)	78 (45.3)
Some support	7 (29.2)	39 (26.4)	46 (26.7)
Limited support	3 (12.5)	21 (14.2)	24 (14.0)
No support	5 (20.8)	19 (12.8)	24 (14.0)
**At most Mild Depression**	14 (60.9)	79 (54.1)	93 (55.0)
**Moderate to Severe depression**	9 (31.9)	67 (45.9)	76 (45.0)

**Table 2:** Chi-square test of association between demographic, clinical, and COVID-19 characteristics and potential MDD.

Variables	At Most Mild Depression	Moderate to Severe Depression	Chi-Square/Fisher Exact	*p*-Value
**Demographic characteristics**				
**Gender**			0.367	0.654
Male	14 (60.9%)	9 (39.1%)		
Female	79 (54.1%)	67 (45.9%)		
**Age categories**			1.014	0.596
≤25	4 (44.4%)	5 (55.6%)		
26–40	36 (52.2%)	33 (47.8%)		
>40	53 (58.2%)	38 (41.8%)		
**Employment status**			8.709	0.004
Employed	92 (57.9%)	67 (42.1%)		
Unemployed	1 (10.0%)	9 (90.0%)		
**Place of employment**			4.301	0.507
School boards	49 (64.5%)	27 (35.5%)		
Healthcare industry	4 (44.4%)	5 (55.6%)		
Keyano college	10 (50.0%)	10 (50.0%)		
Oil Sands industry	6 (50.0%)	6 (50.0%)		
Municipal or Government Agency	8 (66.7%)	4 (33.3%)		
Other	14 (48.3%)	15 (51.7%)		
**Marital status**			0.646	0.737
Married/Partnered/cohabiting	70 (56.9%)	53 (43.1%)		
Divorced/Separated/Widowed	8 (50.0%)	8 (50.0%)		
Single	15 (50.0%)	15 (50.0%)		
**Clinical characteristics**				
**History of Depression from a health professional**				
Yes	16 (29.6%)	38 (70.4%)		
No	77 (67.0%)	38 (33.0%)	20.688	0.000
**History of Bipolar Disorder from a health professional?**				
Yes	3 (50.0%)	3 (50.0%)		
No	90 (55.2%)	73 (44.8%)	0.064	1.000
**History of Anxiety from a health professional?**				
Yes	27 (38.0%)	44 (62.0%)		
No	66 (67.3%)	32 (32.7%)	14.301	0.000
**History of Alcohol Abuse**				
Yes	2 (66.7%)	1 (33.3%)	0.167	1.000
No	91 (54.8%)	75 (45.2%)		
**History of Drug Abuse**			0.021	1.000
Yes	1 (50.0%)	1 (50.0%)		
No	92 (55.1%)	75 (44.9%)		
**History of Personality Disorder**				
Yes	1 (100.0%)	0 (0.00%)		
No	92 (54.8%)	76 (45.2%)	0.822	1.000
**No history of mental health diagnosis**				
Yes	36 (41.4%)	51 (58.6%)		
No	57 (69.5%)	25 (30.5%)	13.501	0.000
**Other mental health diagnosis**				
Yes	8 (50%)	8 (50%)		
No	85 (55.6%)	68 (44.4%)	0.181	0.793
**History of Antipsychotics medications**				
Yes	2 (50.0%)	2 (50.0%)		
No	91 (55.2%)	74 (44.8%)	0.42	1.000
**History of Benzodiazepines**			5.013	0.039
Yes	0 (0.0%)	4 (100.0%)		
No	93 (56.4%)	72 (43.6%)		
**History of Mood Stabilizers**			0.436	0.545
Yes	5 (45.5%)	6 (54.5%)		
No	88 (55.7%)	70 (44.3%)		
**History of Antidepressants**			6.547	0.013
Yes	22 (40.7%)	32 (59.3%)		
No	71 (61.7%)	44 (38.3%)		
**History of Sleeping Tablets**			13.319	0.000
Yes	3 (15.8%)	16 (84.2%)		
No	90 (60.0%)	60 (40.0%)		
**History of other medications**			2.496	0.253
Yes	3 (100.0%)	0 (0.0%)		
No	90 (54.2%)	76 (45.8%)		
**No history of mental health medication**				
Yes	25 (41.0%)	36 (59.0%)		
No	68 (63.0%)	40 (37.0%)	7.610	0.007
**Respondents received mental health counselling in the past year**				
Yes	27 (42.2%)	37 (57.8%)		
No	66 (62.9%)	39 (37.1%)	6.865	0.011
**Respondents would like to receive mental health counselling**				
Yes	33 (37.1%)	56 (62.9%)		
No	60 (75.0%)	20 (25.0 %)	24.481	0.000
**COVID-19 related characteristics**				
**Fearful of contracting the coronavirus during the pandemic**				
Yes	83 (53.5%)	72 (46.5%)		
No	10 (71.4%)	4 (28.6%)	1.659	0.265
**Fearful of their close friends or family members contracting the coronavirus during the pandemic**			5.084	0.033
Yes	87 (53.4%)	76 (46.6%)		
No	6 (100.0%)	0 (0.0%)		
**Friends or family members sick from the coronavirus disease**			0.157	0.733
Yes	66 (55.5%)	53 (44.5)		
No	25 (52.1%)	23 (47.9%)		
**Self-isolate/self-quarantine due to COVID-19 symptoms, recent travel, or because you were in contact with someone who may have COVID-19**			0.347	0.635
Yes	54 (52.9%)	48 (47.1%)		
No	38 (57.6%)	28 (42.4%)		
**Frequency watching television images of sick and dead people caused by coronavirus**			1.004	0.606
Daily	41 (55.4%)	33 (44.6%)		
Less than daily	42 (57.5%)	31 (42.5%)		
Did not watch	10 (45.5%)	12 (54.5%)		
**Frequency reading newspaper and internet articles related to the pandemic**			0.154	0.908
Daily	53 (54.1%)	45 (45.9%)		
Less than daily	38 (56.7%)	29 (43.3%)		
Did not read	2 (50.0%)	2 (50.0%)		
**Lost your job due to the COVID-19**			5.743	0.019
Yes	6 (30.0%)	14 (70.0%)		
No	87 (58.4%)	62 (41.6%)		
**Support from family and friends since the COVID-19 pandemic declared**			12.945	0.004
Absolute support	52 (69.3%)	23 (30.7%)		
Some support	25 (48.1%)	27 (51.9%)		
Limited support	9 (34.6%)	17 (65.4%)		
No support	6 (40.0%)	9 (60.0%)		
**Support from the Government of Canada since the COVID-19 pandemic declared**			6.243	0.099
Absolute support	16 (72.7%)	6 (27.3%)		
Some support	17 (68.0%)	8 (32.0%)		
Limited support	13 (46.4%)	15 (53.6%)		
No support	45 (50.0%)	45 (50.0%)		
**Support from the Government of Alberta since the COVID-19 pandemic declared**			11.483	0.008
Absolute support	13 (81.3%)	3 (18.8%)		
Some support	16 (69.6%)	7 (30.4%)		
Limited support	7 (31.8%)	15 (68.2%)		
No support	55 (52.4%)	50 (47.6%)		
**Support from the employer since the COVID-19 pandemic declared**			14.530	0.002
Absolute support	54 (69.2%)	24 (30.8%)		
Some support	19 (43.2%)	25 (56.8%)		
Limited support	12 (52.2%)	11 (47.8%)		
No support	7 (30.4%)	16 (69.6%)		

**Table 3:** Multivariate logistic regression model for respondents’ likelihood to present with Moderate to Severe MDD.

Variables		Coefficient	Standard Error	Wald Statistic	*p*-Value	Odds Ratio	95% C.I. for Odds Ratio	
							Lower	Upper
Unemployed		2.290	1.369	2.800	0.094	9.876	0.675	144.421
**COVID-19 Related variables**								
Lost job due to the COVID-19 pandemic		1.277	0.794	2.591	0.107	3.587	0.757	16.996
Support from family and friends since the COVID-19 pandemic	Absolute Support			1.531	0.675			
	Some support	0.142	0.501	0.080	0.777	1.152	0.431	3.077
	Limited Support	−0.001	0.688	0.000	0.998	0.999	0.259	3.848
	No Support	0.892	0.775	1.325	0.250	2.440	0.534	11.145
Support from the Government of Alberta since the COVID-19 pandemic	Absolute Support			4.713	0.194			
	Some Support	0.038	0.987	0.002	0.969	1.039	0.150	7.195
	Limited Support	1.660	0.995	2.783	0.095	5.260	0.748	36.980
	No Support	0.578	0.874	0.437	0.508	1.783	0.321	9.891
Support from your employer since the COVID-19 pandemic	Absolute Support			5.836	0.120			
	Partial Support	1.251	0.527	5.638	0.018 *	3.495	1.244	9.818
	Limited Support	0.519	0.633	0.673	0.412	1.680	0.486	5.808
	No Support	0.114	0.721	0.025	0.874	1.121	0.273	4.610
**Clinical Covariates**								
History of Depression		1.535	0.579	7.036	0.008 *	4.643	1.493	14.435
History of Anxiety		0.712	0.546	1.699	0.192	2.039	0.699	5.948
History of taking Sleeping Tablets		1.743	0.851	4.193	0.041 *	5.715	1.078	30.304
Not on any medication for mental health concerns		−0.712	0.623	1.306	0.253	0.491	0.145	1.663
Received mental health counselling in the past year		−0.839	0.571	2.161	0.142	0.432	0.141	1.323
Willingness to receive mental health counselling		1.701	0.527	10.395	0.001 *	5.478	1.948	15.403

* Significance at *p* < 0.05.

## Data Availability

Data associated with this study will be made freely available upon reasonable request to the corresponding author.
